# NLRP-3 Inflammasome: A Key Target, but Mostly Overlooked following SARS-CoV-2 Infection

**DOI:** 10.3390/vaccines10081307

**Published:** 2022-08-12

**Authors:** Consolato M. Sergi

**Affiliations:** 1 AP Division/Pathology Laboratories, Children’s Hospital of Eastern Ontario, University of Ottawa, Ottawa, ON K1H 8L1, Canada; csergi@cheo.on.ca; Tel.: +613-737-7600; Fax: 613-738-4837; 2 Department of Laboratory Medicine and Pathology, University of Alberta, Stollery Children’s Hospital, University Alberta Hospital, Edmonton, AB T6G 2B7, Canada

## 1. Introduction

The last two years have shown many political and scientific debates during the current Coronavirus Disease 2019 (COVID-19) pandemic [1]. Hydroxychloroquine, an antimalarial drug, demonstrated some antiviral mechanisms [2]. Such a drug inhibits inflammatory cytokines such as IL-1, IL-6, and TNF-alpha, with favorable in vitro studies and in vivo studies showing clinical improvements and decreases in viral loads in COVID-19 patients [2]. It is clear and reasonable that during a pandemic, any reduction in hospitalization by prevention instead of cures should have priority. However, the support of a different line of therapy against Severe Acute Respiratory Syndrome, Coronavirus 2 (SARS-CoV-2), aiming at host responses rather than the virus itself, may be beneficial, particularly now that the rate of infection is lower than in the initial stages of the pandemic despite controversies among scientists and public [1,3,4]. It is now well known that four-fifths of the infected population have no symptoms or present mild/moderate symptoms. Most of the remaining 20% of the infected population may or may not experience a severe form of infection with atypical interstitial pneumonia. Substantially, only 1 in 20 people of the 20% infected population will develop acute respiratory distress syndrome (ARDS), which may lead to multi-organ failure [1,5]. Limited lockdowns and restrictions may have been useful for containing the infection at some point of the pandemic in some regions, but mental health issues and economic disasters have not been properly evaluated [6]. The vaccination program has been successful in reducing the number of hospitalizations worldwide [7]. Nevertheless, it seems to reduce the probability of experiencing severe symptoms only and does not eradicate the virus as initially indicated. A few antiviral drugs have been proposed with modest efficacy, and natural compounds have variable effectiveness against SARS-CoV-2 [8,9,10].

On the other hand, there is some growing evidence that targeting the NLRP3 (nucleotide-binding domain leucine-rich repeat [LRR] and pyrin-containing receptor 3) inflammasome is useful and may harbor a solid rationale in the COVID-19 pandemic [11,12,13]. NLRP3 prevents the cytokine storm detected in some individuals infected with SARS-CoV-2 and may be an excellent opportunity for supporting the end of this pandemic. The activation of the NLRP3 inflammasome could participate in the initial phase of the innate immune response during COVID-19 and may be extremely useful for expanding this knowledge. A few drugs have shown a direct or indirect action against this signaling pathway, and the NLRP3 inflammasome could favorably represent a therapeutic target during COVID-19 [5,14,15,16].

NLRP3 is a critical 115 kDa protein. It consists of three domains, including a central oligomerization domain (NOD, nucleotide-binding, and oligomerization domain), a C-terminal LRR (leucine-rich repeat) domain, and an intrinsic N-terminal effector PYD domain (pyrin domain) [17,18]. This last domain can recruit the ASC adapter (Apoptosis-associated Speck-like protein containing a CARD or Caspase recruitment domain). It has been identified that NLRP3 is an extraordinary cytosolic-stress sensor. It can trigger a pro-inflammatory signaling pathway involved in innate immune responses [19,20,21]. In many circumstances, the natural response remains the first line of defense against any infection. NLRP3’s activation is followed by assembling a multiprotein signaling complex in the cytosol. The inflammasome is the name of this powerful complex because of its interaction with NEK7 (NIMA-related kinase 7), which is a protein belonging to NIMA-related kinases and an essential mediator of NLRP3 activation downstream of potassium effluxes [22]. The mammalian NEK group belongs to serine/threonine kinases named NEK1–NEK11, which control numerous aspects of mitotic and non-mitotic functions [23]. Subsequently, the action of recruiting the ASC adapter via its PYD domain, by self-recruiting, is triggered through its CARD domain. In the cytoplasm, the inflammasome concentrates in specks, which are micrometric ultrastructural particles [24,25].

The activation of the NLRP3 inflammasome entails two steps. First, the priming signal activates the nuclear factor kappa-B (NF-κB) pathway. It allows the transcription of NLRP3 and other genes encoding some pro-inflammatory cytokines or interleukins (pro-IL-1β and pro-IL-18). This initial step is triggered by binding microbial or endogenous molecules (pathogen-/damage or endogenous-associated molecular patterns, also shortened as PAMP and DAMP) to their receptors, which are labeled PRRs (pattern recognition receptors). The proper activation or second signal induces the appropriate assembly of the inflammasome [26].

**Figure 1:**
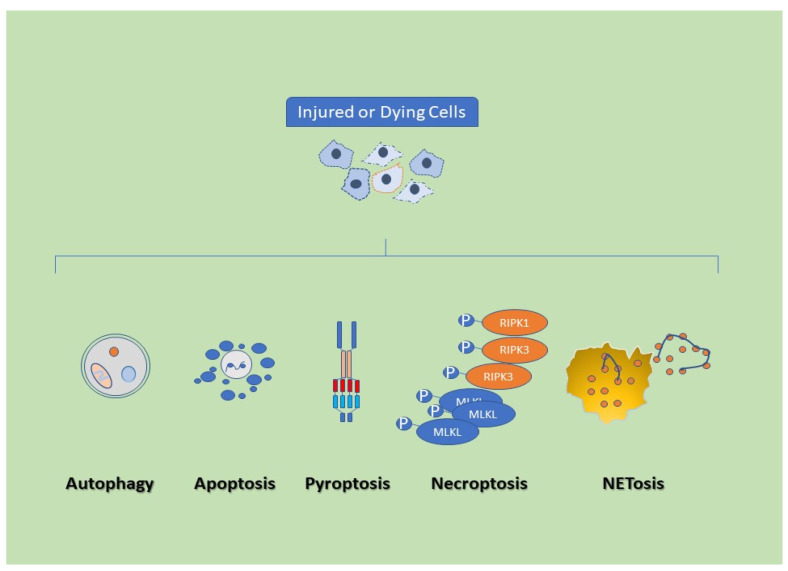
Cells may die from accidental or regulated cell death. Cell death may occur in a setting in which the immune system is notified, triggering immunity against dead-cell antigens. Immunogenic cell death contrasts with silent efferocytosis, in which dying and dead cells are cleared by phagocytosis without any inflammatory or immunologic reactions, as well as with tolerogenic cell death that actively inhibits immune responses. While autophagy and apoptosis both have immunogenic and tolerogenic cell death qualities, pyroptosis, necroptosis, and NETosis seem to demonstrate an exquisite an immunogenic cell death quality. Notes: MLKL, mixed lineage kinase domain; RIPK, receptor interacting protein kinase; P, Phosphate; NET, neutrophil extracellular traps. NETosis is a unique form of cell death. It is featured by the release of modified (decondensed) chromatin. This chromatin is decorated with bactericidal proteins (granules) and released to the extracellular space.

NLRP3 inflammasome relates to incorporating cellular stress signals (e.g., potassium efflux exposure to microbial toxins, crystals, lysosomal rupture, and mitochondrial dysfunction). NLRP3 oligomerizes and recruits the adapter protein ASC, which in turn allows the recruitment and cleavage of pro-caspase-1 to caspase-1. The activated caspase-1 cleaves gasdermin D (GSDMD) and releases its active N-terminal domain (GSDMD-N). It cleaves pro-IL-1β and pro-IL-18 into their active forms, IL-1β and IL-18. The GSDMD-N inserts itself into the plasma membrane to allow the secretion of IL-1β and IL-18. Simultaneously, it induces inflammatory cell death of the cell by pyroptosis, which is only one of the events leading to cell death. Apoptosis is considered “silent” and inhibits immune responses. Conversely, necroptosis and pyroptosis act as “whistleblowers”. They cause the delivery of pro-inflammatory signals into extracellular surroundings (Figure 1).

Substantially, the inflammasome is a transactivation platform for caspase-1. This caspase controls the maturation of the pro-inflammatory interleukins IL-1β and IL-18. Caspase 1 acts by proteolytic cleavage. IL-1β and IL-18 are produced as precursors in the cytosol following cellular stimulations. During viral infections, the NLRP3 inflammasome is activated. The priming signal is then based on the recognition of nucleic acids or proteins of the virus by the innate immunity receptors, the Toll-like receptors (TLR), which stimulate the signaling pathway leading to the activation of NF-κB [27]. The secondary activation or signal is induced by a series of very different events. They include a lysosomal rupture, which causes the dismissal of cathepsin B into the cytosolic environment, by the bouncing production of reactive oxygen species (ROS), the detection of the viral genome via the DHX33 helicase (DEAH-box helicase 33) (also the *Ribonuclease* L.), or ionic imbalances owing to the activity of ion channels, such as P2 × 7 (purinergic receptor P2 × 7), or even the creation of channels formed by viral viroporins [28,29]. At this point, it is essential to reiterate that activating the NLRP3 inflammasome and establishing a pro-inflammatory immune response can be beneficial or harmful depending on the stage of the infection. In a mouse model of Influenza-A virus infection, the late inhibition of the NLRP3 inflammasome by a specific inhibitor, MCC950, prevents the runaway of the immune system and is associated with increased weight and decreased pulmonary inflammation and animal deaths. Conversely, its early inhibition reduces the weight and increases the mortality of infected animals [30].

In COVID-19, several risk factors associated with the most severe forms of COVID-19 linked to the activation of the NLRP3 inflammasome have been identified. They include aging, diabetes mellitus, obesity or overweight, and hypertension [31]. The chronic activation of the NLRP3 inflammasome in these clinical situations is common and can be life-threatening. Diabetic or obese individuals show uric acid, cholesterol crystals, or palmitic acid that cause chronic low-grade inflammation in the body. At this point, the NLRP3 inflammasome joins. This chronic activation inevitably promotes pulmonary fibrosis and restrictive cardiomyopathy, rendering individuals more vulnerable to cardio-respiratory damage associated with COVID-19. Thus, a healthy lifestyle is probably the best assurance against any viral infections and not only SARS-CoV-2 [31]. The progressively increasing age remains the strongest predictor of COVID-19 severity and mortality risk, but the diet is also crucial. In fact, adults with a switch of the immunometabolism from glycolytic to ketolytic patterns experience protection against influenza infection and generic inflammatory damage [32]. An aging model of natural rodent (murine) beta coronavirus (mCoV) infection with mouse hepatitis virus strain-A59 (labeled as “MHV-A59”) demonstrated clearly that mCoV is pneumotropic. It terrifically recapitulates several clinical hallmarks of COVID-19 [32]. Aged mice infected with mCoV-A59 exhibit increased mortality and a higher rate of systemic inflammation in the hypothalamus, heart, and adipose tissue. They also show neutrophilia and loss of γδ T cells in the lower respiratory tract. The utilization of ketogenesis in aged mice promotes tissue protective γδ T cells. Such a ketogenic switch disables the NLRP3 inflammasome. It reduces pathogenic monocytes in the lungs of infected old mice. These data support the ketogenic checkpoint as a potential key to regulating immune responses against coronavirus infection in the elderly population.

Capsid proteins (N proteins) of SARS-CoV-2 can activate the NLRP3 inflammasome, as demonstrated in mice [33] and humans [25,34]. The activation of NLRP3 inflammasome pathways has also been observed ex vivo in circulating monocytes of patients with COVID-19 and in the lung sections taken from patients who died of COVID-19 [34]. Some virus proteins could play a direct role in inflammasome activation. An RNA sequence encoding the protein Spike, rich in guanine and uracil (GU), is recognized by the TLR8 expressed by human monocytes and macrophages. This recognition is at the origin of the transcription of pro-IL-1β and the NLRP3 protein, representing the first signal. The binding of Spike to TLR8 further encourages alternative activations of the NLRP3 inflammasome. Then, it results in the activation of caspase-8 [35]. The SARS-CoV protein E is a viroporin causing calcium efflux from the endoplasmic reticulum and Golgi intermediate compartment (ERGIC) [36]. It has been associated with high concentrations of IL-1β in the lung tissue. The viral genome’s ORF8b, or protein 8b, can activate the NLRP3 inflammasome by directly interacting with its LRR domain [37]. Viroporin 3a encoded by ORF3a is responsible for K+ effluxes into the cell, which triggers the activation of the NLRP3 inflammasome [38,39,40,41]. Moreover, the nucleocapsid would also participate in the activation of the inflammasome by interacting directly with NLRP3 [33]. Finally, the binding of the Spike protein to ACE2 and TLR4 would generate the second activation signal [42]. The N protein of the virus indeed binds directly to GSDMD, thus preventing its cleavage by caspase-1 and its activation. The Spike protein binds to TLR4 and its ACE2 (angiotensin-converting enzyme 2) receptor to allow the formation of NRLP3. The GU (guanine and uracil)-rich sequence of the RNA encoding the Spike protein is recognized by TLR8, inducing the transcription of genes involved in NLRP3 inflammasome signaling by activating the pathway signaling Myd88 (myeloid differentiation primary response 88). TLR8 also induces alternative activations of the NLRP3 inflammasome, involving caspase-8, RIPK (receptor-interacting serine/threonine-protein kinase) 1, and RIPK3. In COVID-19 patients with a severe presentation, several studies have described this increase in concentrations of pro-inflammatory cytokines, including IL-1β, IL-18, IL-1RA (interleukin-1 receptor antagonist), IL-18Bpa (interleukin 18-binding protein), IL-6, IL-2R, IL-8, IL-10, TNF-α (tumor necrosis factor-alpha), and CXCL10 (C-X-C chemokine motif ligand 10), among others [43,44,45,46]. In the lung tissue, IL-1β allows the recruitment of acute inflammatory cells (neutrophils). They produce NETs or neutrophil extracellular traps. NETs are extracellular DNA fibers comprising histone and granule proteins of cytoplasmic origin. NETs represent a primitive form of innate response against pathogens. This response is able to degrade toxic bacterial factors. However, NETs can also create endothelial and epithelial damage in the lung parenchyma, which are underlining risk factors for the development of ARDS. Due to the ubiquitarian expression of ACE2 proteins, SARS-CoV-2 could damage other organs via the activation of the NLRP3 inflammasome in these organs.

Previously, we suggested that an animal model based on the NLRP3 inflammasome may be beneficial in studying COVID-19 [3]. We are familiar with this animal model because of our previous studies on inflammatory bowel disease (IBD) [47,48]. In these models, *Citrobacter rodentium*, which is a non-invasive Gram-negative bacterium, is a natural mouse pathogen. This microorganism is commonly used to investigate enteric infections and bacteria-promoted inflammation as it resembles IBD and enterohaemorrhagic *Escherichia coli* infection in humans [47,48]. TLR-2 and TLR-4, the signaling adaptor protein MyD-88, and NF-κB mediate the inflammatory response to *C. rodentium* by recruiting polymorphonucleated neutrophils and monocytes/macrophages through the progressive induction of numerous chemokines. The same chemokines are critical in the autophagy-inflammasome interplay of heart failure [49]. Intriguingly, COVID-19 patients may also show signs and symptoms that may illustrate a pattern resembling an IBD, at least in some cohorts of these IBD patients [50,51]. In mice, the oral transmission by *C. rodentium* starts with the passing through the cecum and the subsequent colonization of the large-bowel epithelium. It determines the destruction of the brush-border of intestinal microvilli, the depletion of goblet cells, and some epithelial cell changes (hyperplasia). The intestinal homeostasis and epithelial integrity are intimately regulated by other molecules, including the cytosolic nucleotide-binding oligomerization domain (NOD) and the NOD-like receptor (NLR) family expressed in both monocytes/macrophages and epithelial cells. Mice lacking NOD1 or NOD2 are compromised in *C. rodentium* clearance with classical signs of inflammation and dissemination [52]. In macrophages, the NLRP3 protein is a crucial component in the immunologic response to *C. rodentium* [47,53] even though unclarity remains on how the *C. rodentium*-related activation of NLRP3 inflammasome plays. This microorganism triggers procaspase-1 dimerization and, progressively, self-activation, which subsequently processes the maturation of some cellular interleukins (pro-IL-1b and pro-IL-18) to the active cytokines, leading to their secretion.

Moreover, the Gram-negative extracellular enteric microorganism *C. rodentium* can promote caspase-1-dependent IL-1b maturation. It favors this pathway using a synergistic Toll-like Receptor 4 (TLR-4) pathway associated with the activation of the NLRP3 pathway in vivo [25,26]. Rodents lacking the *Nlrp3* gene (the so-called *Nlrp3*^−/−^ mice) show more vulnerability to induced experimental IBD [54,55,56]. Of note, *Nlrp3*^−/−^ monocytes/macrophages did not respond specifically to pathogen-associated microbial patterns Previously, we clearly demonstrated that the advantage of IL-1b in mice requiring the NLRP3 inflammasome might notably boost the net clearance of this Gram-negative extracellular enteric microorganism by encouraging an activation of inflammatory macrophages clearly in the early stages of the intestinal infection. On the other hand, IL-1b overreaction may lead to have a disadvantageous impact in wild-type rodents [47]. Therefore, we fervently advocate that the *Nlrp3*^−/−^ mouse model may be employed as an efficacious and efficient veterinary model to test SARS-CoV-2 countermeasures accurately [3]. To the best of our knowledge, only one research article was triggered by our call in 2021 [3].

Zeng et al. infected THP-1-derived monocytes/macrophages, *NLRP3^−/−^* animals and human ACE2 transgenic mice with live SARS-CoV-2 [57]. The authors found that the NLRP3 inflammasome plays a vital role in the host lung immune response to SARS-CoV-2 invasion. The inhibition of the NLRP3 inflammasome mitigated the release of COVID-19-related pro-inflammatory cytokines in both cell cultures and rodents. The severe pathological pattern caused by SARS-CoV-2 in lung tissues was diminished in *Nlrp3^−/−^* mice compared to wild-type animals (C57BL/6 mice). Finally, the individual inhibition of the NLRP3 inflammasome by MCC950 eased excessive lung inflammation (pneumonia/pneumonitis). There is not much time but targeting the NLRP3 inflammasome is a promising immune intervention against severe COVID-19 cases.

Different treatments targeting events upstream of NLRP3 inflammasome activation or downstream have been assessed for the management of patients with COVID-19 [5]. These treatments, already used or under development for treating other inflammatory diseases, have indeed been repositioned in the context of the management of COVID-19. Chemarin et al. listed colchicine, emricasan, DFV890, and dapansutrile for the inhibition of the activation of NLRP3 inflammasome and canakinumab, anakinra, disulfirame, and dimethylfumarate for the inhibition of the inflammatory cascade promoted by the NLRP inflammasome. However, numerous natural and chemical products may be able to target the NLRP3 inflammasome activation and the subsequent inflammatory cascade.

There is substantial evidence that science has been highly politicized, and some chemical compounds have not been adequately assessed or not properly employed [1,58]. Lucchesi et al. [59] reported on the clinical and biological data on the use of hydroxychloroquine anti SARS-CoV-2, endorsing the exquisite role of the NLRP3 inflammasome in the pathogenetic pathways of respiratory pathology [59]. In our opinion, there is some relatively good evidence that the NRLP3 inflammasome has been mostly overlooked, while being strongly activated in SARS-CoV-2 infection [60]. The reasons may be different, but political leaders supporting some nonconventional (antiviral) drugs may have attracted such attention on themselves and counterproductively allowed some medical communities to be ostracized by other government adherent political figures and medical colleges leading to a frank sabotage of the scientific freedom. The ARDS progressing with systemic inflammation has a tremendous and inexorable lung injury [61,62,63]. It is associated with the release of cytokines with inflammatory character. These cytokines include IL-1β and IL-6, and an equivalent cytokine storm is notably detected in the severe infection of SARS-CoV-2 [64,65]. There is an evident dysregulation of pro-inflammatory cytokines and their cascade. It is triggered by an intense and rapid activation of the human innate immune response. In particular, the aberrant IL-6 production is incredibly predictive of COVID-19 fatality, as clearly shown following the postmortem evaluation of COVID-19 patients [66]. Although the inflammatory inherent foundation underlying COVID-19 death rate states the expansion of immunoregulatory agents of paramount importance, there is almost certainly no fully satisfactory supportive animal model in which some medications can adequately and safely be investigated. Of note, it has been indicated that the NLRP3 inflammasome may also be deactivated by dapsone, a sulfone used in combination with clofazimine and rifampicin to treat leprosy [12], but the processes by which the NLRP3 inflammasome is involved in COVID-19 and its severity remain unclear and requires more investigation.

Some other NLRP3 inflammasome inhibitors using natural compounds are probably crucial to point out here. Isoandrographolide, which targets the NOD-like Receptor (NLR), harbors a cellular differentiation-promoting and hepatoprotective effect [67]. This compound inhibits the activation of the NLRP3 inflammasome activation. It attenuates silicosis in mice [67]. Mulberroside is one of the leading bioactive constituents in mulberry (*Morus alba* L.) [68]. It targets the TNF-α receptor and tyrosinase. Mulberroside A promotes the decrease in the expression of IL-1β, IL-6, and TNF-α and inhibits the activation of NLRP3, caspase-1, and NF-κB as well as the phosphorylation of extracellular signal-regulated kinase (ERK), c-Jun N-terminal kinase (JNK), and p38, exhibiting anti-inflammatory other than antiapoptotic effects [68]. Muscone is a compound that is well known in traditional Chinese medicine [69,70,71,72]. Muscone inhibits the activation of the NLRP3 inflammasome and the promotion of NF-κB. It targets the TNF-α receptor, IL-6 receptor, NF-κB, as well as the NOD-like Receptor (NLR). Muscone remarkably reduces inflammation and ultimately improves cardiac functions and survival rates. Licochalcone is a compound removed from the root of the *Glycyrrhiza* [73], and Licochalcone B inhibits amyloid β self-aggregation and reduces metal-induced Aβ42 aggregation through chelating metal ions. Moreover, it inhibits the phosphorylation of NF-κB and p65 in the LPS signaling pathway. Licochalcone B explicitly inhibits the NLRP3 inflammasome by disrupting NEK7-NLRP3 interactions [74]. Ruscogenin, a steroid sapogenin arisen from *Ophiopogon japonicus*, consistently targets the NOD-like receptor [75,76,77]. Ruscogenin attenuates ischemia-induced blood–brain barrier dysfunction in the brain by suppressing NLRP3 inflammasome activation and the MAPK pathway. Moreover, ruscogenin exerts significant anti-inflammatory and anti-thrombotic activities [75,76,77]. A sequiterpene lactone, the arglabin, targets NOD-like Receptor (NLR), farnesyl transferase, and autophagy [10,78,79,80,81,82]. Arglabin, or better ((+)-Arglabin as chemically correctly identified), is a natural compound derived from *Artemisia glabella*. It is an NLRP3 inflammasome inhibitor. Arglabin shows anti-inflammatory and antitumor activities as well. 4′-Methoxyresveratrol is a polyphenolic compound isolated from *Dipterocarpaceae*, with antiandrogenic, antifungal, and anti-inflammatory activities [83]. 4′-Methoxyresveratrol alleviates age-induced inflammation by suppressing MAPK/NF-κB signaling pathway and the activation of the NLRP3 inflammasome [84]. Soyasaponin II is a triterpenoid saponin with antiviral activities. Soyasaponin II inhibits the replication of herpes simplex virus (HSV) 1, cytomegalovirus (CMV), influenza virus (*Orthomyxoviridae*), and human immunodeficiency virus (HIV) 1 [85]. Soyasaponin II serves as an inhibitor for Y-Box Binding Protein 1 (YB-1) phosphorylation and *nlrp3* inflammasome priming in rodents against Lipopolysaccharide/D-galactosamine (LPS/GalN)-induced acute liver failure. Picroside II is an iridoid compound that belongs to a large group of monoterpenoids. It is derived from *Picrorhiza*. It exhibits anti-inflammatory and anti-apoptotic activities. It enhances immune function by inhibiting the activation of NLRP3 inflammasome and NF-κB pathways [86,87]. Picroside II is an antioxidant with impressive activity by lowering ROS assemblies.

In conclusion, we need to work hard, be humble, and show data exactly. From memory, face masks were not advocated for more than six months by major institutions and national agencies. The starting recommendation by the World Health Organization (WHO) was that only individuals who are sick and symptomatic and individuals who are taking care of subjects with potential COVID-19 should wear masks [88,89]. In reviewing the chronology of the COVID-19 pandemic, we can see how politics drives science and science drives politics as well, and the degree of ambiguities in statements is still impressive [90]. Ambiguity and attitudes harbor a detrimental effect on individuals and their mental health [91]. About six months later, both the Center of Disease Control (CDC), United States of America, and WHO updated their recommendations that facemasks should be used in public places where physical distancing is challenging to maintain [92]. It would have been relevant to have it in place at least at the beginning of the pandemic to reduce the number of infections considering the knowledge on coronaviruses [93]. Now, the prolonged deprivation of exposure to microorganisms of the immune system in children and adults may be a component of acute hepatitis of unknown origins, AHUO [94]. Finally, there is enough and compelling literature to support the use of several molecular compounds targeting the NLRP3 inflammasome for COVID-19 patients considering the current relative mild symptomatology of the O-micron variant and subvariants. The *Nlrp3*^−/−^ rodent with adequate veterinary pathology support is a critical animal model for properly studying SARS-CoV-2 infections, aiming to clarify the numerous obscure aspects of this infection.

## Data Availability

All articles mentioned in this editorial are publicly available (e.g., PubMed).
